# The Effects of Bisphenol A on Human Male Infertility: A Review of Current Epidemiological Studies

**DOI:** 10.3390/ijms241512417

**Published:** 2023-08-04

**Authors:** Mafalda Presunto, Melissa Mariana, Margarida Lorigo, Elisa Cairrao

**Affiliations:** 1 FCS-UBI, Faculty of Health Sciences, University of Beira Interior, 6200-506 Covilhã, Portugal; a38823@fcsaude.ubi.pt (M.P.); melissa.r.mariana@gmail.com (M.M.); margarida.lorigo@gmail.com (M.L.); 2 CICS-UBI—Health Sciences Research Centre, University of Beira Interior, 6200-506 Covilhã, Portugal

**Keywords:** bisphenol A, endocrine disruptor, reproductive toxicity, infertility, male reproduction

## Abstract

Endocrine disruptor chemicals (EDCs) can have a harmful effect on the human body’s endocrine system and thus adversely affect the development, reproduction, neurological, cardiovascular, and immune systems and metabolism in humans and wildlife. According to the World Health Organization, EDCs are mostly man-made and found ubiquitously in our daily lives, notably in pesticides, metals, and additives or contaminants in food and personal care products. Human exposure occurs through ingestion, inhalation, and dermal contact. Bisphenol A (BPA) is a proven EDC capable of mimicking or blocking receptors and altering hormone concentrations and metabolism. Although consumed in low doses, it can stimulate cellular responses and affect the body’s functions. In humans, exposure to BPA has been correlated with the onset or development of several diseases. This literature review aimed to verify the effects of BPA on human male infertility using the most recently published literature. Thus, this review allowed us to conclude that this compound seems to have harmful effects on human male fertility, causing changes in hormonal and semen characteristics. However, these conclusions lack more robust and reproducible scientific studies. Even so, and since male infertility prevalence is increasing, preventive measures must be taken to ensure male fertility.

## 1. Introduction

Endocrine disruptor chemicals (EDC) can have a harmful effect on the endocrine system [1]. They are defined by the Environmental Protection Agency of the United States of America as “an agent that interferes with the synthesis, secretion, transportation, binding or elimination of natural hormones in the body that are responsible for the maintenance of homeostasis, reproduction, development and/or behaviour” [2,3]. By interfering with the hormonal system, they adversely affect the development, reproduction, neurological, cardiovascular, immune, and metabolism systems in both humans and wildlife. These effects can be observed after long exposure and may also have consequences for the next generation, i.e., they may vary depending on the time of exposure and the hormonal balance of the exposed individual, which is determined by age, sex, and other factors [1].

According to the World Health Organization (WHO), EDs are mostly man-made and are found in various materials such as pesticides, metals, and additives or contaminants in food and personal care products. Human exposure occurs through ingesting food, dust, and water and inhaling gases and particles in the air and through the skin. There may also be passage to the foetus through the placenta and to children through breast milk [1]. More specifically, adults come into contact mainly through ingestion of contaminated drinking water, meat, and fatty dairy products and inhalation of polluted air, while babies are exposed through breastfeeding, contact with baby products, and also by inhalation of polluted air [2].

EDs are highly heterogeneous and can be classified as natural and synthetic or grouped according to their origin [2,4]. They are also considered “chemical chameleons”, i.e., they have different mechanisms of action depending on the value of their concentration and the stage of development of the affected tissue [5]. More concrete examples of these endocrine disruptors are diethylstilbestrol, Bisphenol A, phthalates, polybrominated diphenyl ethers, and parabens, among others [1].

Bisphenol A (BPA) is an endocrine disruptor capable of mimicking or blocking receptors and altering hormone concentrations and their metabolism. Despite being consumed in low doses, it can stimulate cellular responses and affect body functions [6]. In humans, increased BPA levels correlate with the onset or development of various diseases, health problems, and medical conditions [7]. It can have several effects throughout the body and at different stages of the life cycle [8]. 

BPA seems to cause multi-systemic and multi-organ toxicity in animal models; however, several questions about the effects of exposure in humans still need to be answered, and more research is needed [9]. Thus, this substance represents an important challenge for the current industrialised society, requiring an assessment of the overall impact on human health since we are continuously exposed to this compound.

The available literature points out that the reproductive system may be one of the main targets of BPA toxicity, which manages to interfere negatively with male fertility. Thus, this literature review seeks to gather, update, and summarise the existing information on the effects of this substance on human male infertility.

## 2. Physical and Chemical Properties of Bisphenol A

**Figure 1:**
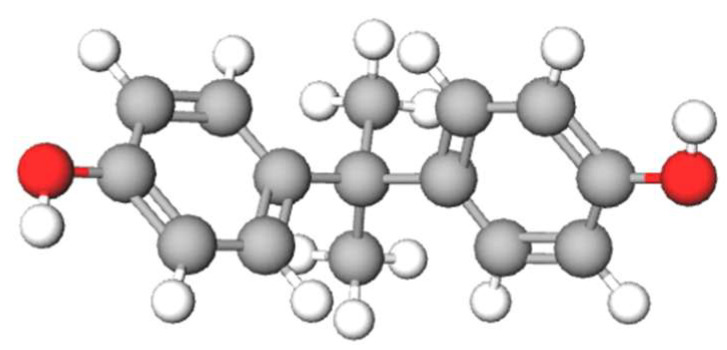
Chemical structure of bisphenol A.

BPA, also designated by IUPAC as 4-[2-(4hydroxyphenyl)propan-2-yl]phenol, is an endocrine disruptor that has estrogenic activity [10]. It was synthesised for the first time by the Russian scientist Aleksandr P. Dianin in 1891 and later investigated in the 1930s during the search for synthetic oestrogens [8]. It is a synthetic organic compound, C_15_H_16_O_2_, which belongs to a group of phenols and has hydroxyl residues directly attached to the aromatic ring (Figure 1) [11,12]. 

At room temperature, BPA is a solid, white, crystalline substance with a mild odour that has a molecular weight of 228.29 g/cm^3^, a melting point of 156 °C, and a boiling point of 220 °C (at a pressure of 5 hPa). In addition, it has an octanol–water partition coefficient of log Pow = 3.32, indicating that it has a good solubility in fats and, therefore, a low solubility in water (~200 mg/dm^3^ at 25 °C). The presence of hydroxyl groups determines their good reactivity. Like other phenols, BPA can be converted into ethers, esters, and salts [11].

BPA does not occur freely in nature, and when exposed to air, it undergoes photo-oxidation and degradation, resulting in a low half-life (0.2 days) [6].

## 3. Production and Application

The presence of BPA in the environment is linked only to anthropogenic activity, being considered one of the most produced chemicals worldwide [8,11]. Global BPA production gradually increased from 5 to 8 million tons between 2010 and 2016 and is estimated to reach 10.2 million by 2023. There has been a prominent increase in production in the emerging markets of China, India, Russia, and Mexico [6]. Due to its mass production, many derivatives of this substance are released into the environment, thus contributing to an increase in pollution and contamination of soils and groundwater [13].

In the 1950s, it was observed that BPA could be polymerised to make polycarbonate plastic, a miraculous product that is cheap, lightweight, transparent, and colourable; resistant to impact, heat, and chemicals; unalterable over time; and easy to mould and thermoform [14]. The manufacturing process involves a reaction between acetone and two phenols in a continuous, closed process under certain temperature conditions using an acid catalyst. The substitution of atoms in different numbers and positions results in the formation of analogous compounds with specific characteristics intended for various industrial applications [15].

It is currently used as a material to produce phenol resins, polyacrylics, and polyesters, but it is mainly to produce epoxy resins and polycarbonate plastics. Epoxy resins are used as coatings on canning cans and metal bottle covers, as protective coatings and finishes, in automobile parts, adhesives, and aerospace applications, and as a coating for PVC pipes. Polycarbonate plastics are used to manufacture automotive lenses, household appliances, food packaging, plastic bottles, pacifiers, and even toys [10]. BPA has also been used since the 1960s as a component of many dental restoration materials [16]. Recently, its uses have expanded to producing optical and electronic materials [17].

Despite all the applications, consumers’ concern about the possible consequences for humans has resulted in “BPA free” products and the development of bisphenol analogues to replace BPA. These compounds (e.g., bisphenol S, bisphenol F, bisphenol AF, bisphenol E, and many others) have been extensively used in numerous everyday products, and humans are persistently exposed to these compounds after they are released from the products. Unfortunately, these other derivatives also seem to have similar harmful effects, and some of them are even more toxic. Specifically, bisphenol S and bisphenol F were found to have similar hormonal activity as BPA (estrogenic, antiestrogenic, androgenic, and antiandrogenic activities) and also alter organ weights, reproductive endpoints, and enzyme expression both in vitro and in vivo [18,19,20]. Thus, BPA analogues have also been considered as EDCs and may pose a human health risk similar to BPA.

## 4. Sources of Exposure

**Figure 2:**
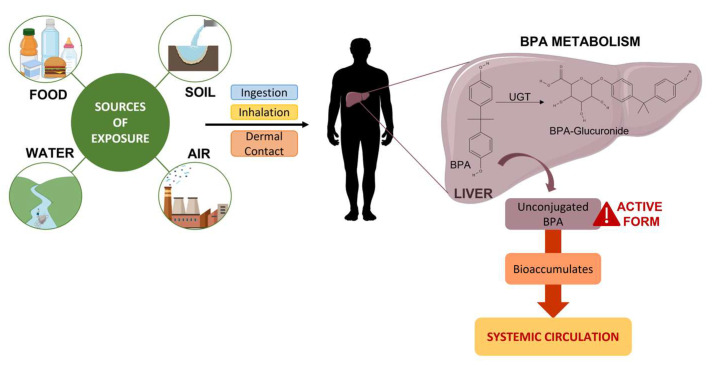
Sources of exposure and consequent metabolism of BPA.

The degree of exposure to BPA can vary dramatically depending on various socioeconomic factors, lifestyle, comorbidities, and routes of exposure, like food, water, air, and soil (Figure 2) [11].

### 4.1. Food

The main source of BPA contamination in humans is through food [9,10,21]. Its presence in edible products is related to the exposure of animals and raw materials to BPA, the accumulation of BPA in the environment, and the contact of food with polymers containing this substance [11]. Direct contamination of food is caused by the migration of BPA present in the containers in which food is stored. Epoxy resins produced with BPA are used as a coating on the surfaces of metal cans that are in contact with food and beverages, while polycarbonate plastics are used in containers that transport/store food and beverages [10]. The European Food Safety Authority (EFSA) has recently updated the tolerable daily intake of BPA from 4 μg to 0.2 ng per kilogram of body weight per day [22,23]. The previous TDI was temporary due to uncertainties in the evidence, whereas the revised EFSA decision was made according to new scientific evidence that established BPA as a health risk concern since, in all age groups, average and high exposure to BPA exceeded the 0.2 ng per kilogram of body weight per day [23]. On the other hand, the Environmental Protection Agency of the United States of America sets the tolerable value daily at 50 μg/kg/day [24]. Moreover, according to the European Commission, the migration of BPA into food from varnishes or coatings applied to materials and objects should not exceed a specific migration limit of 0.05 mg of BPA per kilogram of food. However, the migration of BPA is not permitted from varnishes or coatings applied to materials and articles specifically intended to be in contact with infant formulae, processed cereal-based foods, baby foods, special medical foods developed to meet the nutritional requirements of infants and young children, and milk beverages and similar products specifically intended for young children, as referred to in Regulation (EU) No 609/2013 [22,23]. BPA can migrate from plastics after washing and sterilisation in alkaline solutions or in hot water (e.g., steam) [10]. The migration ratio of BPA from polycarbonate bottles to other solutions varies according to their chemical properties. Temperature and, particularly, the prolonged use of bottles increases the hydrolysis of the polymer, causing a more intense migration to water [11]. BPA is authorised to be used as a monomer in plastic food contact materials in accordance with Commission Regulation (EU) No 10/2011. In addition, Commission Implementing Regulation (EU) No 321/2011, which amends the abovementioned agreement, imposes a restriction on the use of BPA in the manufacture of baby bottles for children, in accordance with the precautionary principle [22,23].

### 4.2. Water

BPA can be found in the aquatic environment and has been detected at high levels in leachates from landfills or factories, especially those that manufacture and process plastics [10]. In surface waters, it is usually found in low concentrations; however, near waste deposits contaminated with this substance or waste deposits of plastic materials, the concentrations are much higher [11]. BPA can also be detected in river waters; it can be degraded in aerobic conditions but not in anaerobic conditions. Despite the degradation of BPA in freshwater by bacteria and sunlight, its half-life of 3 to 5 days may be sufficient to cause harmful effects on aquatic organisms due to the metabolites that persist. Interestingly, BPA can persist longer in seawater than in river water without any degradation (about 30 days), and the bio-available fraction of dissolved BPA can increase with salinity. Thus, the possibility of contamination of a marine organism may be greater than a freshwater organism. Therefore, from the aquatic environment, the ingestion of freshwater fish or seafood contaminated with BPA may be the main route of contamination for humans [10,25]. Bacteria and other microorganisms that effectively degrade this substance may be useful in its removal from the polluted environment [11].

### 4.3. Air

Inhalation is the second main source of exposure [9]. BPA is emitted into the atmosphere during industrial activity, where it can remain due to its adhesion to particulate matter. The possibility of inhaling high levels of BPA through the air is very low [6,10,11]. However, due to the large quantities produced annually, workers in companies engaged in the production of plastics are an exception [16]. Various household items manufactured from BPA, such as epoxy-based flooring and electronic equipment, can release and volatilise this compound with prolonged use. Consequently, BPA can accumulate in household dust and be inhaled [9].

### 4.4. Soil

The main source of BPA in soils is the terrestrial application of sewage or bio-solid sludge and human waste. BPA released into groundwater or surface water can be absorbed into soil or sediment. It was found that there are higher levels of BPA in sediments than in surface waters. However, soil contamination can be correlated with population density due to the increase in human waste contaminated with BPA, such as household and/or industrial waste [10,25].

BPA is thought to have a moderate affinity with soil organic matter and thus is unlikely to be mobile or bio-available in soils. However, mobility can be affected by soil chemistry and texture. There is evidence of an increase in BPA sorption in the presence of iron, cadmium, and lead and rapid and complete desorption in sandy and acidic soils [25].

## 5. Conjugation, Metabolism, and Excretion

In humans, orally ingested BPA is rapidly and efficiently absorbed through the gastrointestinal tract (>95% of the dose) and subsequently metabolised in the liver, which plays an essential role in the metabolism of this substance (Figure 2) [8,9,26]. Its glucuronidation, the main biotransformation pathway, is a detoxification reaction that is carried out by uridine diphosphate glycuronosyltransferase (UGT), a glycosyltransferase that is present in the liver. UGT in the human foetal liver is present at a concentration five times lower than in the adult liver; this means that a foetus may face a higher risk of deleterious effects [9,10]. This process increases the solubility of BPA in water, which leads to faster excretion in the urine (half-life of 5.4–6.4 h) [27]. 

BPA glucuronide, with little or no estrogenic activity, is the main metabolite found in urine, while free or unconjugated BPA, considered the active form, is the main component present in faeces [10,26]. More than 99% of free BPA and its metabolites are excreted in faeces and urine, and less than 1% accumulates in tissues [10]. Some studies suggest that there may be gender differences in metabolite concentrations in the urine, with women having higher levels of BPA sulphate and men having higher levels of BPA glucuronide [8,10]. 

BPA is also conjugated in vivo in BPA sulphate by sulfotransferases found in the liver. BPA sulfation eliminates its estrogenic activity [8,26]. 

However, unlike dietary exposure, almost all BPA resulting from transdermal exposure prevents hepatic metabolism, resulting in significantly higher concentrations of the unconjugated form (free BPA) in the bloodstream [27].

### Detection in Biological Samples

Given the prevalence of BPA in our environment, it is not surprising that measurable levels have been detected in most individuals who were analysed. It is possible to detect the presence of BPA in human tissues and fluids, including maternal blood (0.3–18.9 ng/mL), maternal urine (31.9 μg/L), amniotic fluid (median = 0.26 ng/mL), placental tissue (median = 12.7 ng/g), umbilical cord blood (0.2–9.2 ng/mL), breast milk (0.61–0.7 μg/L), and human colostrum (3.41 ng/mL) [7,11,16]. Concentrations of unconjugated BPA in human serum have been detected at levels ranging from 0.2 to 20 ng/mL [16]. Depending on the pathway by which the individual is exposed, BPA levels in the circulation may also be different [8]. As most of the BPA consumed orally is excreted in the urine (concentration varies between 1.6 and 946 μg/L) in less than 24 h after consumption, and the expected concentrations in the blood are very low, urine is the preferred body fluid for estimating BPA exposure in humans [26].

Several studies have examined BPA levels in the serum of pregnant women, umbilical cord blood, and foetal plasma. The results of these studies indicate that this substance crosses the placental barrier [28]. Of particular concern are the high levels detected mainly in developmental stages with a possible higher sensitivity to BPA [16]. BPA can accumulate in foetuses due to lower metabolic clearance. After swallowing amniotic fluid, BPA can be conjugated by the foetal liver. Thus, a foetus is susceptible to BPA exposure throughout development and may be exposed to even higher levels than those measured in the adult’s blood. Another important consideration for the health of the newborn is the potential exposure to BPA in breast milk since it is a compound with lipophilic properties [16]. By being lipophilic, it can accumulate in adipose tissue, including fat mammary glands, and cause dysregulation in various metabolic processes, both in the baby and in the adult [9,11].

## 6. Mechanisms of Action

BPA, with a structure similar to 17β-oestradiol (E2), binds to both the alpha (ERα) and the beta oestrogen receptors (ERβ), with approximately 10 times more affinity for ERβ. This EDC can also bind to the G-protein-coupled oestrogen receptor (GPER), the gamma peroxisome proliferator-activated receptor (PPAR-γ), and the orphan nuclear oestrogen gamma receptor (ERR-γ) [29]. Binding to these receptors can lead to other changes in cells and tissues, not just endocrine disruptions [27].

It is now widely accepted that BPA not only has the effectiveness of E2 but is also potent in relation to several of its effects, that is, it has a potent estrogenic effect [8]. In addition, it can act as an anti-oestrogen, blocking the oestrogen response by competing with endogenous E2. It can also bind directly to androgen receptors (AR), possibly being anti-androgenic, blocking endogenous androgen action [30]. 

Endogenous oestrogens have various effects at the biological level, from every organism to the systems of organs, cells, and gene expression [8]. They play a key role in the testicle since their biosynthesis occurs in the testicular cells. The absence of ERs causes adverse effects on spermatogenesis and steroid synthesis. Physiological levels of E2 are essential for normal spermatogenesis, and when altered, it can lead to the occurrence of pathologies. For example, in infertility, there is an excess of E2 together with a decrease in testosterone [31]. In addition to its estrogenic activity, there is some evidence that BPA binds to the thyroid hormone receptor, acting as an antagonist and preventing the binding of T3 [8]. The affinity for the thyroid receptor is lower than the affinity for the oestrogen receptor, suggesting that elevated BPA levels would be needed to antagonise the hormonal action of the thyroid [7]. 

**Figure 3:**
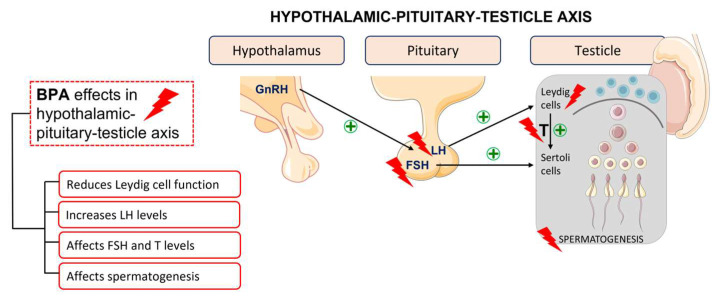
BPA effects in the hypothalamic–pituitary–testicle axis.

Regarding the male reproductive system, this substance perturbates the hypothalamic–pituitary–testicle axis and modulates gene expression and enzymatic activity of testicular steroidogenesis (Figure 3). It is also associated with a decrease in the activity of the antioxidant system, resulting in an increase in oxidative stress, the most common cause of sperm damage [19]. It exerts multidirectional effects as it interacts with several receptors, generates reactive oxygen species, alters cell signalling, causes mutagenic changes, and inhibits DNA methylation [11].

Although the mechanisms of action are not yet fully understood, BPA also interferes with other functions of the body, including the development of the central nervous system, the endocrine action of the pancreas, the immune system, and the function of sex hormones, insulin, leptin, and adiponectin. These BPA effects may also be responsible for the development of several types of cancer, such as breast, prostate, pancreas, pituitary, cerebellar cortex, and heart cancer [11,30].

## 7. Methods

**Figure 4:**
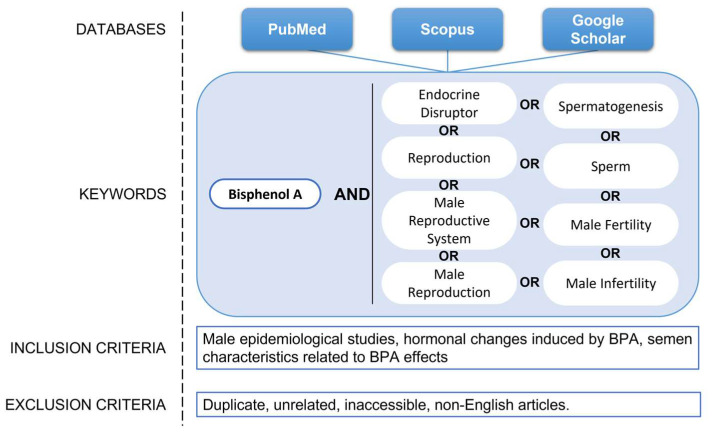
Search strategy with the terms used to search the online databases.

For the preparation of this review, a bibliographic search was conducted from September 2022 to January 2023, using the search databases PubMed, Scopus, and Google Scholar. The following items were used as keywords: (bisphenol A) AND (endocrine disruptor); (bisphenol A) AND (reproduction); (bisphenol A) AND (male reproductive system); (bisphenol A) AND (male reproduction OR male fertility OR male infertility); (bisphenol A) AND (spermatogenesis OR sperm). Priority was given to articles from the last ten years to study the consequences of BPA on male infertility; however, older articles that were considered relevant were not excluded. The inclusion criteria comprised epidemiological studies regarding male infertility that related BPA to hormonal changes and semen characteristics. The exclusion criteria included all articles that were duplicates, unrelated, inaccessible, and not written in English. The search strategy is described in Figure 4.

## 8. Effects on Human Male Fertility

Widespread and ongoing exposure to BPA in humans was confirmed using biomonitoring studies in the general population, in which more than 80% of participants had detectable levels in the urine [32]. Analysis of data from the available literature indicates that the reproductive system is one of the targets of BPA toxicity. This substance acts mainly by mimicking the effect of oestrogen, modifying DNA methylation, and modulating enzymatic activities, resulting in metabolic diseases, spermatogenesis defects and/or infertility in men. The deleterious effect on male reproductive function can occur during embryonic, pubertal, and/or adult life [33]. 

The disrupting endocrine effects of BPA on male reproductive health have raised concerns over the past few decades. Studies report that exposure to this compound is associated with reduced sperm count, worsening of sperm motility and speed, decreased epididymis weight, poor insulin signalling and glucose homeostasis, and decreased testicle steroids, follicle-stimulating hormone (FSH), and testosterone (T) levels in the serum. However, these observations are based on animal models and in vitro studies [34,35,36,37,38]. Regarding the effects of BPA on the human male reproductive system, studies are still limited, and the underlying molecular mechanisms remain uncertain.

Infertility is one of the main health problems that affect human sociocultural life and is defined, according to the WHO, as “the inability to achieve a clinical pregnancy after 12 months or more of regular unprotected sexual intercourse” [39]. There are two types: primary infertility, which refers to couples who have never had a child, and secondary, which refers to infertile couples who have already managed to become pregnant at least once [40]. It affects 8 to 15% of couples worldwide, with an expected tendency to increase in the coming years, and about 50% of cases are attributed to male factors [33]. Risk factors for male infertility include varicocele, ageing, sexually transmitted diseases, lifestyle factors, and prolonged or intense exposure to certain types of toxins, chemicals, radiation, medications, and environmental toxicants [41,42]. All these factors can lead to a low count (oligospermia) or absence of sperm (azoospermia) and also to several morphological changes [42].

This medical condition is related to dysfunction of the pituitary gland and male gonads. Testicular functions include testosterone production and spermatogenesis, both being regulated by the hypothalamic–pituitary–gonad axis through a negative feedback system. This axis is further influenced by other endocrine organs, such as the adrenal gland and adipose tissue [43]. 

During spermatogenesis, gametes are created daily in the seminiferous tubules. All stages of the process are regulated by the endocrine system, specifically by T, E2, FSH, and luteinizing hormone (LH). The mechanisms that lead to male infertility are not completely clear, although changes in hormonal balance, particularly during sperm development, may affect sperm quality [44]. Its dysfunction, triggered by endocrine disruptors such as BPA, can result in the arrest or alteration of spermatogenesis, negatively affecting sperm production and quality [33,34]. Spermatogenesis is a complex process that involves genetic, hormonal, and environmental factors, and the failure of any of these can result in infertility. Recent studies have shown a decline in semen quality in men from several countries, including developing and developed countries, again emphasizing the role of environmental toxicants as a cause for this phenomenon [45]. 

In this section, we seek to find the possible connection of BPA exposure with several aspects of male infertility, incorporating data from epidemiological studies.

### Evidence from Epidemiological Studies

In the testes, steroid hormones play an important role in spermatogenesis, semen production, and maintenance of secondary sexual characters and libido. They are also a target for endocrine disruptors [46]. Considering the endocrine disruption properties of BPA, an increase in its exposure in men may be associated with altered hormone levels and, therefore, with possible reproductive dysfunction [47]. In relation to the studies that characterise the parameters of semen quality, an analysis of the spermatogram or semen allows for evaluating the function of the testicle and has a prognostic value in the evaluation of fertility. The spermatogram is the cornerstone in the evaluation of male infertility [43].

In studies performed using animal models, BPA was suggested to be a testicular toxicant since it decreased sperm quality and motility, caused oxidative stress, and altered steroid synthesis [34,35,36,37,38]. Regarding the effects of BPA in humans, the data are still inconsistent, with limited evidence and no proven causality. Most of the studies observed an association between BPA exposure and sperm parameters, which may be related to male infertility. However, it is important to note that most of the studies are related to occupational and high levels of exposure [7,34]. 

The studies presented in this review are grouped according to evaluated parameters and main conclusions, including those with alterations in hormone levels, sperm parameters, hydroxymethylation, or no association. Note that in serum, the normal reference values are as follows: FSH of 1.5–12.4 mIU/mL, LH of 1.2–7.8 mIU/mL, TT of 2.5–8.4 pg/mL, prolactin of 2–18 ng/mL, and E2 of less than 40 pg/mL [48].

Since 2013, several studies have investigated the associations between BPA exposure in adulthood and fertility in different populations of men (e.g., occupational exposure, fertile or potentially infertile population, general population, and young people unaware of their fertility), but the results remain contradictory and inconclusive [49]. As in vivo human exposure is not feasible to assess the effects of BPA on male fertility due to ethical issues, the available evidence comes from observational epidemiological studies [50].

Three different studies evaluated occupational exposure to BPA in Chinese populations. The first, a cross-sectional study conducted by Zhou et al., evaluated the association between serum BPA levels and sex hormones. The sample included a total of 290 men, of whom 137 worked in a factory where they were exposed to BPA and 153 worked in a different factory not exposed to BPA. They found that increased serum BPA concentration was associated with a decreased free androgen index (FAI) and androstenedione (AD) and FT levels and increased sex hormone binding globulin (SHGB) levels [51]. In the second study, each of the 559 workers enrolled provided serum samples and were divided into two groups: exposed and unexposed to BPA. Both serum BPA concentrations and detection rates were higher in exposed men than in unexposed men, although the two groups showed no differences in serum levels of SHBG, total testosterone (TT), or inhibin B or AD. When comparing different durations of exposure (less and more than 5 years), a longer exposure was associated with increased serum BPA and SHBG levels and decreased AD levels. Briefly, chronic occupational exposure to BPA is associated with altered levels of male sex hormones and may affect male reproductive health [47]. Similar hormonal changes were also detected in a study by Liu et al. that recruited 592 workers, with and without occupational exposure to BPA, and collected urine and blood samples. The authors concluded that high exposure to BPA is associated with increased levels of prolactin, E2, and SHGB and a decrease in FSH, AD, and FAI [52]. In a similar perspective, a Japanese case-control study aimed to evaluate exposure to bisphenol A diglycidyl ether in epoxy resin workers. The results showed that bisphenol A diglycidyl ether can generate BPA endogenously with higher BPA levels in the urine of the epoxy resin workers when compared to controls. Furthermore, the exposed workers had decreased FSH levels, while there was no difference between LH and FT levels compared to the controls [53]. These studies presented similar results, with increased BPA exposure being related to hormonal level alterations including, specifically, increased SHBG and decreased AD, FAI, and FSH, which may contribute to male infertility. 

Using a representative sample of the general Danish population, Lassen et al. evaluated urine, semen, and blood samples from 308 men. The authors concluded that BPA exposure is associated with higher concentrations of T, LH, E2, and FT, as well as a lower percentage of progressive sperm. However, no association was detected for other specific parameters of semen [32]. Similar results were found in an Italian population, where increased urinary BPA levels were positively related to increased TT levels, but no relation was found for E2 [54]. In a more recent study, Liang et al. collected urine and blood samples from 560 men, aged between 18 and 55, recruited from a low-industrialised mountainous area of China. The authors investigated whether exposure to environmental BPA at a lower level was related to serum concentrations of gonadotropic hormones. The results showed that exposure to BPA is associated with increased serum LH and FSH levels in male smokers along with decreased serum TT levels in men with BMI > 25 kg/m^2^. These findings suggest that the effects of environmental exposure to BPA on hormone levels may be modified by tobacco smoke and BMI [55]. These differences might be due to the different populations analysed as well as their specific characteristics, including age, BMI, and tobacco.

From a different perspective, Joensen et al. investigated the association between BPA exposure and testicular function in young Danish men with (n = 65) and without (n = 130) mutations in the filaggrin gene (FLG). Within the FLG mutation carrier group, higher urinary BPA concentrations were found to be associated with higher serum T and E2 levels and lower FSH levels. Furthermore, those with higher urinary BPA values had lower sperm motility. None of these associations were evident in the control group. The changes found in testicular function were not only due to the differences in BPA exposure levels since urinary levels did not differ between the two groups [56]. 

An investigation by Den Hond et al. aimed to verify whether greater exposure to endocrine disruptors was associated with an increase in subfertility in men, for which they used semen, urine, and blood samples from 120 men from four fertility clinics in Belgium. The authors divided the men into two groups according to total motile count (TMC) (cases: TMC < 20 million and controls: TMC > 20 million) and concluded that increased BPA levels were associated with a decrease in serum T and a higher risk of subfertility [57]. On the other hand, in a study on fertile men (n = 375), exposure to low environmental levels of BPA was suggested to be associated with a modest reduction in markers of free testosterone (FT), but any effects on reproductive function were considered to be small and of uncertain clinical significance [58].

More recently, a cross-sectional study also evaluated the association between urinary BPA concentration, semen quality, and reproductive hormone levels. The sample population included 215 healthy young university students (18–23 years old) from Spain [49], in which there was a reduction in the capacity of Leydig cells (increased LH levels) and a decrease in sperm count. If confirmed, these findings suggest that environmental BPA levels may negatively affect endocrine function in young people. However, no significant associations were found between BPA and other semen parameters or levels of reproductive hormones, contrary to the studies cited above [49]. Using the same cohort, during the Murcia Young Men’s Study (Spain), 158 healthy participants (18-23 years old) were gathered to evaluate urinary BPA concentrations and the sperm DNA fragmentation (SDF) index. There was no association between urinary BPA concentrations and the SDF index in the total group. However, in the subgroup of men with an SDF index > 30%, positive associations were observed. The authors reported that, if confirmed, the results obtained suggested that environmental levels of BPA may have a negative impact on sperm function [59]. 

From the studies presented so far, despite some contradictory results, it is possible to assume that exposure to BPA may alter the concentration of several hormones, thus affecting male reproductive function, which can culminate in infertility.

Some investigations were conducted to analyse BPA concentrations and semen parameters in men already diagnosed with infertility. In a study involving 149 couples from an infertility clinic undergoing their first or second intracytoplasmic sperm injection procedure (ICSI), urinary BPA concentration in male partners and its relationship with semen quality parameters were evaluated. The authors found an association between increased BPA concentration and lower sperm count, sperm concentration, and sperm vitality [60]. After examining 190 men recruited from an infertility clinic, Meeker and co-workers also showed a decrease in sperm concentration and motility and morphology changes associated with urinary BPA concentration [61]. Similar results were also found in men (n = 218) exposed to BPA in the workplace, with decreased sperm concentration, total count, vitality, and motility; however, there was no association with morphology [62].

La Rocca et al. analysed BPA levels in the serum of 70 infertile and 83 fertile men from Italian metropolitan, urban, and rural areas. The purpose was to assess whether residential areas, with different expected levels of BPA, had different outcomes of potential infertility. The results showed that, when compared to the participants of urban and rural areas, infertile men from the metropolitan area had significantly higher levels of BPA, which were directly correlated with increased gene expression of ERα, ERβ, AR, aryl hydrocarbon receptor (AhR), and pregnane X receptor (PXR). These findings reinforce the concept that the living environment determines the main pattern of exposure to endocrine disruptors in the population and that the nuclear receptors panel in peripheral blood mononuclear cells is a potential biomarker that can be used to assess the impact of ED on reproductive health [63]. However, in a Chinese less industrialised area with lower exposure to environmental contaminants, BPA was related to decreased sperm quality. The aim of that cross-sectional study, which was conducted by Ji et al., was to examine the association of urinary BPA concentrations and sperm parameters related to movement. The participants included 500 fertile men from China who provided urine for BPA measurement and semen for analysis with a computer-aided sperm analysis (CASA) system. From the results obtained, the authors concluded that environmental exposure to BPA in a less industrialised area, where the levels of BPA in urine are lower, is related to decreased sperm concentration and motility parameters and increased velocity rates [64]. Scientific knowledge indicates that semen quality is a dominant component in determining male fertility. Current evidence from in vitro and human studies [65,66,67] show that a reduction in spermatozoa curvilinear velocity (VCL) and the amplitude of lateral head displacement (ALH) and an increase in linearity (LIN) are related to decreased male fertility. Thus, BPA-induced reductions in sperm concentration and ALH and increases in LIN can lead to a deterioration of male fertility. Changes in spermiogenesis and poor sperm movement may explain male subfertility resulting from BPA exposure, although the underlying mechanisms are still unknown [64].

In a different study, Omran and colleagues aimed to identify any kind of association between urinary BPA levels and infertility-related factors in Egyptian male patients. The study population consisted of 50 infertile men and 50 healthy men as controls. Semen quality parameters (concentration, count, morphology, and motility) and oxidative stress were determined along with sperm DNA damage. Urinary BPA concentrations were found to be similarly increased both in the cases and controls, with a negative association with semen quality and antioxidant levels and a positive association with DNA damage [68]. Also investigating an Egyptian population, a case-control study was performed to evaluate the association between BPA levels and male fertility. After collecting urine and semen samples from all the participants, BPA levels, semen parameters, and hormone profiles were analysed. In addition to BPA concentrations being significantly elevated in infertility cases compared to controls, the results also showed a correlation between those levels and sperm motility and some reproductive hormones (FSH, LH, TT, and E2) [48]. These results are in agreement with those from a study by Lassen et al., where higher levels of LH, TT, and E2 were also found in Danish men [32]. 

In a cross-sectional study on 984 Chinese men from an infertility clinic, there was an association between higher exposures to BPA and deterioration in semen quality, i.e., concentration and total sperm count and progressive and total motility below the normal reference [69]. Increased concentrations of BPA were also observed in infertile men recruited from a reproductive endocrinology unit in Greece. In that study, 55 men with different causes of infertility were analysed for BPA concentrations, and the results were compared to 25 fertile men from the general population. However, there was no difference in BPA concentrations between infertile men and control or between the causes of infertility. Therefore, these results suggest that although male infertility cannot be attributed to BPA exposure, high concentrations of BPA may contribute to this cause [70].

Vitku et al. determined the levels of BPA and oestrogens in the blood plasma and seminal plasma of 174 men with different degrees of infertility, according to their spermatogram. There was a change in E2 metabolism and sperm count, confirming the existence of an inverse association between the concentration of seminal BPA and sperm quality [31]. Later, the same research group also analysed the effects of BPA on spermatogenesis and steroid synthesis. Using the same biological matrices, the authors found that the presence of increased BPA levels in the seminal plasma can contribute negatively to sperm quality, decreasing their number, concentration, and morphology. In addition, the results indicated that BPA influences the synthesis of gonadal and adrenal steroids in several stages, reduces the rate of testicular steroidogenesis, and increases the rate of synthesis in the adrenal gland. Further disruption of steroidogenesis by BPA possibly occurs at the level of E2 metabolism through suppression of its catabolism or through stimulation of aromatase expression and/or activity [46].

In a different manner, during a study including 315 Polish men under the age of 45, Radwan and colleagues analysed the relationship between BPA exposure and semen quality, sperm DNA damage, and aneuploidy. The population sample was recruited from a reproductive health clinic and had normal sperm concentrations. The study provided evidence that BPA exposure is associated with poorer semen quality, considering the observed increase in immature sperm, decrease in sperm motility, and dissection of sperm chromosomes [39]. On the other hand, after evaluating the quality of semen in relation to the concentration of BPA, Pollard and colleagues concluded that higher exposure to this substance is associated with abnormal morphology of the sperm tail; however, no association was found for BPA exposure regarding sperm count. In the investigation, a sample of 161 men aged between 18 and 40 years and with unknown fertility was recruited [71].

With the aim of analysing exposure to BPA and sperm DNA methylation changes, Chinese men occupationally exposed (n = 77) and not exposed (n = 72) to BPA were recruited for sample collection (urine, blood, and semen). It was concluded that exposed men had lower sperm concentration when compared to the control group, and higher urinary BPA levels were associated with a lower degree of sperm DNA methylation [72]. Similarly, Tian and collaborators used semen samples from 158 Chinese factory workers, of whom 72 had exposure to BPA and 86 had no occupational exposure. The data obtained indicated that occupational exposure to BPA is associated with changes in the hydroxymethylation of human semen, which may be one of the possible mechanisms underlying the adverse effects induced by BPA on male reproductive function [73]. In a different study, BPA exposure was also analysed for its association with sperm acetylcholinesterase gene hydroxymethylation. In that comparative study, 74 and 83 male workers in factories with and without occupational exposure, respectively, were selected to enrol and provide urine and semen samples. Increased exposure to BPA was found to be related to increased hydroxymethylation levels, which led the authors to conclude that BPA may have adverse effects on male fertility [74].

A different approach was conducted by Palak et al., who evaluated the association between BPA levels, steroid hormone concentration, and circulating miRNA levels. The researchers found the following associations: increased levels of BPA and reduced E2 and AD concentrations in the seminal plasma of azoospermic men compared to healthy controls; a negative correlation between BPA levels and sperm concentration and normal semen morphology; a significant positive correlation between BPA levels and miR-let-7a and miR-let-7c levels; and a negative correlation with miR-518f levels. These results led the authors to conclude that BPA can negatively affect sperm quality and act directly on seminal plasma, affecting the testicular environment [75].

Although most of the studies described show an association between BPA exposure and changes in male hormones or semen parameters, there are others that did not find any association. Contrary to the studies cited above, Chen et al., who investigated whether exposure to phenols was related to idiopathic male infertility in the general population, concluded that there was no evidence of the association between BPA exposure and male infertility [76].

In the same line of results also appears the LIFE study that aimed to estimate the association between BPA and 35 parameters of semen quality among men of reproductive age from Michigan and Texas, USA. The 418 men recruited from the general population were to avoid the clinical and/or professional context, as observed in many studies already conducted and previously mentioned. This exploratory analysis found no evidence corroborating an adverse relationship between total urinary BPA concentration and semen quality [77].

Interestingly, a study by Hu and colleagues evaluated the interaction between BPA exposure and obesity in semen quality, a factor that increases BPA toxicity in spermatogenesis. The sample consisted of 357 subfertile men recruited from hospitals in the city of Nanjing, China. However, opposite to some studies previously mentioned, there was no relationship between BPA levels and sperm parameters, including sperm concentration, total count, motility, and semen volume [78]. In another investigation including 146 couples undergoing in vitro fertilisation, BPA concentrations did not significantly affect sperm concentration or motility parameters [79].

Based on the Fetal programming of Semen Quality (FEPOS) cohort, Benson and colleagues hypothesised that BPA and its substitutes (BPS and BPF) were related to poor semen quality. After analysing urine and semen samples from 556 young participants (18–20 years), the authors found no association between bisphenol urinary levels and semen characteristics [80]. Similarly, no statistically significant association was found between BPA concentration in the urine and parameters of seminal fluid in 105 men attending a fertility centre [81]. Taking into account that these results are contradictory to many previous investigations, the authors of these studies considered that the lack of association was possibly due to the decreased levels of BPA exposure. 

Most recently, an investigation with the aim to understand the possible differences in exposure to phthalates and BPA between two male populations with different degrees of fertility concluded that there were no differences between urinary BPA levels in both groups. This population consisted of 155 men with changes in fertility and 211 controls, recruited in Milan, Italy [44].

From a different perspective, considering that pregnancy is one of the most vulnerable and sensitive periods, prenatal exposure to environmental chemicals has been widely studied and related to adverse health effects in the life of the mother and the offspring. EDCs can interfere with hormone homeostasis and signalling pathways that withstand pregnancy physiological changes, thus increasing maternal susceptibility and entailing risks not only for the mother but also for the newborn during childhood, adolescence, and adulthood [28]. Thus, two studies investigated maternal BPA exposure and reproductive male function. The Western Australian Pregnancy Cohort (Raine) Study is a large cohort designed to evaluate the effects of prenatal events on child and adult health. In this case, Hart and colleagues used this cohort to analyse how maternal BPA exposure affected male reproductive function later in life (20–22 years). The results showed a weak association between prenatal BPA exposure and testicular function, sperm concentration, and motility [82]. Similarly, in a Danish birth cohort study including 101 young men (18–20 years), there was also a positive association between high maternal exposure to BPA and reduced Leydig cell function; however, no association was found between anogenital distance and semen quality [83].

Overall, despite some contradictory results, most studies show that there is indeed a relationship between increased levels of BPA and semen quality, either directly or indirectly through changes in hormone levels. However, more studies are needed to understand the mechanisms involved in the contribution of BPA exposure to male infertility. Table 1 lists all the epidemiological studies mentioned above and their general conclusions.

## 9. Conclusions

With the industrialisation of modern society and the need for more convenient and practical solutions to keep up with the hectic lifestyle of today’s families, plastic has become the preferred material for food and water containers. Human exposure to BPA is currently of great concern because this chemical has become so widespread in the environment and because it is detected in human biological fluids. Given the numerous sources and endocrine disruption potential of this chemical, it seems advisable to introduce worldwide biomonitoring in order to assess the risk to human health in detail. This monitoring can be a valuable tool in studies looking for relationships between BPA exposure and the prevalence of certain hormonal disorders.

**Figure 5:**
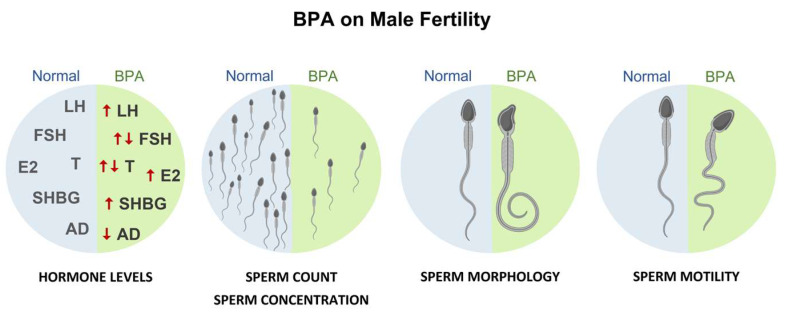
Effects of BPA on male fertility outcomes including normal hormone levels and semen parameters vs. BPA exposure.

The harmful effects on male fertility have been clearly illustrated in animal-based studies; however, human studies remain uncorroborated. In hormonal studies, the endpoints measured were typical: testosterone, oestradiol, follicle-stimulating hormone, luteinizing hormone, sex hormone-binding globulin, and/or androstenedione. In many of the studies analysing hormone levels, high exposure to BPA was associated with lower levels of testosterone compared with the normal reference values and with the respective controls. It was also related to changes in other hormone levels that may also contribute to male infertility. Human studies suggest that exposure to this substance is associated with changes in hormone levels and decreased sperm quality (motility, concentration, count, and vitality) and increased DNA damage and changes in sperm morphology, especially when there is high occupational exposure (Figure 5). 

However, the research available so far is insufficient to draw a definitive conclusion about BPA exposure and its effects on human male infertility. This lack of consensus and the contradictory results are because existing studies are limited, with mostly cross-sectional designs, small sample sizes, variety in groups of men (genetic predisposition, environmental conditions), and other methodological weaknesses. No type of epidemiological study is free of limitations, for example, cohort studies are expensive and require follow-ups, case-control studies are susceptible to information bias and have several external variables, and cross-sectional studies are subject to the time of data assembly, and causality cannot be assessed [84]. The majority of the studies discussed used a cross-sectional design, and there is a great lack of experimental approaches, thus, there is an urgent need to combine prospective cohorts, which encompass a large sample size, and experimental studies. Despite being detected in different biological samples, urine has the most advantages since it is easy to collect and non-invasive and has higher volume and BPA concentrations. However, BPA is rapidly metabolised and excreted in the urine, and although a single sample may reflect prolonged exposure, more samples per participant and throughout a period of time should be used as a strategy to obtain more reliable results. Furthermore, variety in the population samples is also a weakness. In addition to presenting different genetic predispositions and daily life habits, the studies group men from a large age range and not by age group, considering that hormone levels vary over age.

Thus, further studies that overcome these weaknesses are needed to establish the adverse effects of BPA more precisely and to ascertain whether the findings found using in vitro studies and with animal models can also be verified with the same significance in men. It is necessary and extremely important to carry out more homogeneous studies to obtain more consistent and reliable data. The current ambiguous conclusions could be resolved if BPA’s exact mechanism of action were known, which remains to be determined. It would also be interesting to try to devise efficient therapeutic strategies to reverse the negative impacts of BPA on reproduction; for this, it is recommended to study all possible targets (proteins) and their impacts. 

Still, the importance of reducing BPA levels in the environment should be emphasized to safeguard male fertility. Workers exposed to BPA should increase their personal protection (e.g., wearing a mask, gown, etc.) and pay attention to personal hygiene (e.g., not eating in the workplace, bathing after leaving work, etc.) to avoid as much as possible high exposure in the body. World governments should take measures (setting concentration limits, eliminating the use of certain substances, and tight control of agents already identified as causing reproductive harm) to ensure the precautionary principle. Furthermore, new and safer substitutes should also be created and tested, considering that those already replacing BPA have been found to have endocrine-disrupting properties as well, thus imposing a similar or even greater health risk as BPA.

Given the large-scale production of BPA, its constant presence in our daily lives, and the presumed effect on male fertility, measures should be taken to replace it with innocuous components and restrict its use. 

In this review, only BPA is addressed; however, many other endocrine disruptors are ubiquitous in the environment and coexist in our body, which can have potentially synergistic effects on the quality of semen and reproductive hormones. The widespread use of bisphenols is undoubtedly dangerous for testicular function, and a reduction in their use is necessary to preserve male sexual and reproductive health.

## Figures and Tables

**Table 1:** Epidemiological studies, main evaluation characteristics, and general conclusions.

Study	Country	Population Sample Characteristics (Number, Mean Age, Mean BMI, Progeny)	Biological Samples	Evaluated Parameters	General Conclusions	Author, Year Reference
Cross-sectional	China	137 men with and 153 men without occupational exposure (<30–>50 years old)	Blood	Reproductive hormones	Decrease AD, FT, and FAI Increase SHGB	Zhou et al.—2013 [51]
Case-control	China	281 men with occupational exposure (34.12 years, 27.54 kg/m^2^) 278 men without occupational exposure (32.78 years, 26.41 kg/m^2^)	Blood: - Exposed workers—median [BPA] 18.75 ng/mL; - Unexposed workers—median [BPA] 3.37 ng/mL.	Reproductive hormones	Exposure >5 years—increased SHBG and decreased AD No changes between groups in SHBG, TT, or inhibin B or AD	Zhuang et al.—2015 [47]
Cross-sectional	China	165 men with and 427 men without occupational exposure (31.7 years)	Blood Urine: - Exposed workers—median [BPA] 685.9 µg/g Cr; - Unexposed workers—median [BPA] 4.2 µg/g Cr.	Reproductive hormones	Increased prolactin, E2, and SHGB Decrease FSH, AD and FAI	Liu et al.—2015 [52]
Cross-sectional	Japan	42 men (37 years) with and 42 men (38 years) without occupational exposure	Blood Urine: - Exposed workers—median [BPA] 1.06 mmol/mol Cr; - Unexposed workers—median [BPA] 0.52 mmol/mol Cr.	Reproductive hormones	Decreased FSH No association with LH and FT	Hanaoka et al.—2002 [53]
Cross-sectional	Denmark	308 young men from the general population (≈ 18 years)	Semen Blood Urine (median [BPA] 3.25 ng/mL)	Characteristics of semen Reproductive hormones	Increase T, LH, E2, and FT Decreased sperm motility No evidence for other semen parameters	Lassen et al.—2014 [32]
Cross-sectional	Italy	334 men from the general population (20-74 years old, BMI: < 18.5–> 35 kg/m^2^)	Blood Urine (geometric mean [BPA] 4.02 ng/mL)	Reproductive hormones	Increased TT No association with E2	Galloway et al.—2010 [54]
Cross-sectional	China	560 men (32.2 years, BMI: < 18.5–> 25 kg/m^2^)	Blood Urine (geometric mean [BPA] 0.44 µg/L)	Reproductive hormones	Increased LH and FSH in smokers Total T decreased in BMI >25 kg/m^2^	Liang et al.—2017 [55]
Case-control	Denmark	65 young men with FLG mutation 130 controls (19 years, 23 kg/m^2^)	Semen Blood Urine (median [BPA] 3.5 ng/mL)	Characteristics of semen Reproductive hormones	FLG mutation carriers—increased T and E2 and decreased FSH Decreased sperm motility	Joensen et al.—2018 [56]
Case-control	Belgium	120 men from 4 fertility clinics 40 cases (31.6 years, 26.1 kg/m^2^) 80 controls (34.1 years, 24.6 kg/m^2^)	Semen Blood Urine: - Cases—geometric mean [BPA] 1.7 µg/L; - Controls—geometric mean [BPA] 1.5 µg/L,	Reproductive hormones	Decreased T and increased risk of subfertility	Den Hond et al.—2015 [57]
Cross-sectional	USA	375 fertile men from a prenatal clinic (31.9 years, 28.3 kg/m^2^)	Semen Blood Urine (geometric mean [BPA] 1.5 µg/mL)	Characteristics of semen Reproductive hormones	Modest FT decrease No association with semen features or reproductive hormones	Mendiola et al.—2010 [58]
Cross-sectional	Spain	215 healthy college students (median age of 20.4 years, median BMI of 23.7 kg/m^2^)	Semen Blood Urine (geometric mean [BPA] 2.3 ng/mL)	Characteristics of semen Reproductive hormones	Increased LH Decreased sperm concentration No evidence for other parameters of semen or other reproductive hormones	Adoamnei et al.—2018 [49]
Cross-sectional	Spain	158 university students (median age of 20.4 years, median BMI of 23.6 kg/m^2^)	Semen Urine (mean [BPA] 2.05 μg/g)	Sperm DNA fragmentation	Increased SDF index in the SDF subgroup>30%	Kiwitt-Cardenas et al.—2021 [59]
Prospective Cohort	Slovenia	149 men from couples undergoing IVF or ICSI treatment (34.05 years, 27.45 kg/m^2^)	Semen Urine (geometric mean [BPA] 1.55 ng/mL)	Characteristics of semen	Decreased sperm count, sperm concentration, and vitality	Knez et al.—2014 [60]
Cross-sectional	USA	190 men from a fertility clinic (36.4 years, 27.3 kg/m^2^)	Semen Urine (geometric mean [BPA] 1.4 ng/mL)	Characteristics of semen	Decreased sperm concentration and motility Morphology changes Increased DNA sperm damage	Meeker et al.—2010 [61]
Cohort Case-control	China	218 men with and without occupational exposure (<25–>45 years)	Semen Urine	Characteristics of semen	Decreased concentration, total count, vitality, and sperm motility No association with morphology changes	Li et al.—2011 [62]
Case-control	Italy	70 infertile men and 83 fertile men from: Metropolitan (37.2 years, 25.0 kg/m^2^) Urban (34.02 years, 25.2 kg/m^2^) Rural (35.6 years, 26.3 kg/m^2^)	Semen Blood: - Infertile—mean [BPA] 9.3 ng/mL; - Fertile—mean [BPA] 5.7 ng/mL.	Gene expression nuclear receptors	Increased gene expression ERα, ERβ, AR, PXR, and AhR	La Rocca et al.—2015 [63]
Cross-sectional	China	500 fertile men (18–55 years, BMI: < 18.5–> 25 kg/m^2^, ≥1 child)	Semen Urine (geometric mean [BPA] 0.38 µg/L)	Characteristics of semen	Decreased sperm concentration and motility Increased sperm velocity rates	Ji et al.—2018 [64]
Case-control	Egypt	50 infertile men 50 fertile men (20–54 years old)	Semen Urine: - Infertile—median [BPA] 24.2 μg/L; - Fertile—median [BPA] 20.9 μg/L.	Characteristics of semen	Decreased semen quality	Omran et al.—2018 [68]
Case-control	Egypt	100 infertile men (33.53 years, BMI < 30 kg/m^2^) 50 fertile men (35.20 years, BMI < 30 kg/m^2^)	Semen Urine: - Infertile—mean [BPA] 10.07 ng/mL; - Fertile—mean [BPA] 1.69 ng/mL.	Characteristics of semen Reproductive hormones	Increased BPA levels in infertility cases compared to controls Association between sperm motility and FSH, LH, TT, and E2 levels	Shokry et al.—2020 [48]
Cross-sectional	China	984 men from a reproductive health clinic (32.0 years, 23.3 kg/m^2^)	Semen Urine (median [BPA] 2.24 µg/L)	Characteristics of semen	Decreased concentration and total sperm count and progressive and total motility	Chen et al.—2022 [69]
Cross-sectional Case-control	Greece	55 infertile men: - 23 non-obstructive azoospermia (34.6 years); - 20 varicocele (37.4 years); - 12 cryptorchidism (33.7 years). 25 fertile men (30.7 years)	Semen Blood: - Infertile—median [BPA] 0.19 ng/mL; - Fertile—median [BPA] 0.18 ng/mL.	BPA concentration	Very high concentrations only in infertile men, but no differences in concentration between the two groups	Mantzouki et al.—2019 [70]
Cross-sectional	Czech Republic	174 men with several degrees of infertility (35.97 years, 27.32 kg/m^2^)	Semen and blood [BPA]: - Fertile—66 and 47 pg/mL; - Slightly infertile—144 and 137 pg/mL; - Moderately infertile—132 and 114 pg/mL; - Severely infertile—179 and 33 pg/mL.	Characteristics of semen	Decreased sperm count and concentration	Vitku et al.—2015 [31]
Cross-sectional	Czech Republic	191 men with various degrees of infertility: - Fertile (35.9 years, 27.7 kg/m^2^); - Slightly infertile (35.7 years, 26.9 kg/m^2^); - Moderately infertile (35.8 years, 26.1 kg/m^2^); - Severely infertile (35.2 years, 26.4 kg/m^2^).	Semen and blood [BPA]: - Fertile—0.075 and 0.029 ng/mL; - Slightly infertile—0.130 and 0.059 ng/mL; - Moderately infertile—0.153 and 0.072 ng/mL; - Severely infertile—0.158 and 0.019 ng/mL.	Characteristics of semen	Decreased concentration, total count, vitality, and sperm morphology Gonadal and adrenal steroidogenesis changes	Vitku et al.—2016 [46]
Cross-sectional	Poland	315 men from a reproductive health clinic (32.14 years, 26.8 kg/m^2^)	Semen Urine (geometric mean [BPA] 1.84 µg/L)	Characteristics of semen	Decreased sperm motility Increased immature sperm Sperm chromosomal disomy	Radwan et al.—2018 [39]
Prospective Cohort	USA	161 men (28.5 years, 27.1 kg/m^2^)	Semen Urine (geometric mean [BPA] 2.50 ng/mL)	Characteristics of semen	Changes in morphology (sperm tail) No evidence for other semen parameters	Pollard et al.—2019 [71]
Case-control	China	77 men with and 72 men without occupational exposure (22–50 years old)	Semen Urine: - Exposed workers—mean [BPA] 36.23 µg/g Cr; - Unexposed workers—mean [BPA] 1.38 µg/g Cr.	Characteristics of semen Semen methylation	Decreased sperm concentration and methylation	Miao et al.—2014 [72]
Case-control	China	72 men (34.1 years) with and 86 men (34.4 years) without occupational exposure	Semen Urine: - Exposed workers—geometric mean [BPA] 158.41 µg/g Cr; - Unexposed workers—geometric mean [BPA] 0.84 µg/g Cr.	Semen hydroxymethylation	Changes in semen hydroxymethylation	Tian et al.—2018 [73]
Case-control	China	74 men with and 83 men without occupational exposure (<29–>36 years)	Semen Urine: - Exposed workers—geometric mean [BPA] 199.13 µg/g Cr; - Unexposed workers—geometric mean [BPA] 0.77 µg/g Cr.	Sperm hydroxymethylation	Increased BPA levels related to increased sperm acetylcholinesterase hydroxymethylation	Song et al.—2019 [74]
Case-control	Poland	50 men, normospermic (31.30 years, 23.92 kg/m^2^) 46 men, oligoasthenoteratozoospermic (31.36 years, 23.69 kg/m^2^) 20 men, non-obstructive azoospermic (31.20 years, 23.3 kg/m^2^)	Semen	Characteristics of semen Reproductive hormones MiRNA levels	Decreased sperm concentration and morphology Decreased E2 and AD Increased miR-let-7a and miR-let-7c and decreased miR-518f	Palak et al.—2021 [75]
Case-control	China	877 idiopathic infertile men (28.5 years, 23.47 kg/m^2^) 713 fertile men (29.83 years, 23.87 kg/m^2^)	Semen Urine: - Infertile—geometric mean [BPA] 0.612 ng/mL; - Fertile—geometric mean [BPA] 0.621 ng/mL.	Characteristics of semen	No evidence of an association between BPA exposure and male infertility	Chen et al.—2013 [76]
Prospective Cohort	USA	418 men from couples trying to get pregnant (31.7 years, 29.5 kg/m^2^)	Semen Urine (geometric mean [BPA] 0.55—0.67 ng/mL)	Characteristics of semen	No evidence of an association between BPA exposure and semen quality	Goldstone et al.—2015 [77]
Cross-sectional	China	357 subfertile men (28.7 years, 23.39 kg/m^2^)	Semen Urine (geometric mean [BPA] 0.52 ng/mL)	Characteristics of semen	No evidence of an association between BPA exposure and male infertility	Hu et al.—2017 [78]
Prospective Cohort	Republic of Korea	146 men from couples undergoing IVF (36.3 years, 25.9 kg/m^2^)	Blood Urine (geometric mean [BPA] 1.1 ng/mL)	Characteristics of semen	No evidence of an association between BPA and sperm motility and concentration	Kim et al.—2021 [79]
Cross-sectional	Denmark	556 young men (≈19 years old, BMI: <18.5–>25 kg/m^2^)	Semen Urine (median [BPA] 1.30 ng/mL)	Characteristics of semen	No evidence of an association between BPA exposure and semen quality	Benson et al.—2021 [80]
Cross-sectional	Italy	105 men from a fertility clinic (40.5 years, BMI: 18.5–>25 kg/m^2^)	Semen Urine (mean [BPA] 0.24 µg/g Cr)	Characteristics of semen	No evidence of an association between BPA exposure and semen quality	Caporossi et al.—2020 [81]
Case-control	Italy	155 infertile men (40.4 years) and 211 fertile men (36.1 years) (BMI: <18.5–>25 kg/m^2^)	Semen Urine: - Infertile—mean [BPA] 0.47 µg/g Cr; - Fertile—mean [BPA] 0.69 µg/g Cr.	Characteristics of semen	No evidence of an association between BPA exposure and semen quality	Caporossi et al.—2022 [44]
Prospective Cohort	Australia	705 young men and available prenatal maternal serum samples (20–22 years old, ≈24 kg/m^2^)	Semen Blood	Characteristics of semen Reproductive hormones	Weak association between testicular function, sperm concentration, and motility No association between testicular volume, testicular and pituitary hormones, and total sperm output	Hart et al.—2018 [82]
Prospective Cohort	Denmark	101 young men and prenatal maternal serum samples (median age of 19.3 years, median BMI of 21.2 kg/m^2^)	Semen Blood	Characteristics of semen Testicular function	Reduced Leydig cell function No association between anogenital distance and semen quality	Holmboe et al.—2022 [83]

## Data Availability

Not applicable.
